# The rate of W chromosome degeneration across multiple avian neo-sex chromosomes

**DOI:** 10.1038/s41598-024-66470-7

**Published:** 2024-07-17

**Authors:** Hanna Sigeman, Philip A. Downing, Hongkai Zhang, Bengt Hansson

**Affiliations:** 1 Department of Biology, Lund University, Ecology Building, 223 62 Lund, Sweden; 2 Ecology and Genetics Research Unit, University of Oulu, Oulu, Finland

**Keywords:** Sex chromosome, W chromosome degeneration, Sylvioidea, Neo-sex chromosome, Rate of gene loss, Molecular evolution, Evolutionary biology

## Abstract

When sex chromosomes evolve recombination suppression, the sex-limited chromosome (Y/W) commonly degenerate by losing functional genes. The rate of Y/W degeneration is believed to slow down over time as the most essential genes are maintained by purifying selection, but supporting data are scarce especially for ZW systems. Here, we study W degeneration in Sylvioidea songbirds where multiple autosomal translocations to the sex chromosomes, and multiple recombination suppression events causing separate evolutionary strata, have occurred during the last ~ 28.1–4.5 million years (Myr). We show that the translocated regions have maintained 68.3–97.7% of their original gene content, compared to only 4.2% on the much older ancestral W chromosome. By mapping W gene losses onto a dated phylogeny, we estimate an average gene loss rate of 1.0% per Myr, with only moderate variation between four independent lineages. Consistent with previous studies, evolutionarily constrained and haploinsufficient genes were preferentially maintained on W. However, the gene loss rate did not show any consistent association with strata age or with the number of W genes at strata formation. Our study provides a unique account on the pace of W gene loss and reinforces the significance of purifying selection in maintaining essential genes on sex chromosomes.

## Introduction

Sex chromosomes have evolved from autosomes numerous times in animals and plants^1–4^. Male heterogametic systems (XY systems) are more widespread than female heterogametic systems (ZW systems), and in both these systems the sex chromosome pair often (but not always) evolve recombination suppression and genetic differentiation^1,2^. Theory predicts that the absence of recombination on the sex-limited chromosome (Y or W) will result in degeneration, primarily due to reduced efficacy of purifying selection^5,6^. In animals, this process has been studied empirically mainly using evolutionarily old and highly degenerated Y chromosomes, for example in eutherian mammals and fruit flies^7,8^. The Y chromosomes in both groups have experienced heavy gene decay, through loss-of-function mutations and/or complete deletion of the DNA sequence.

Ancient Y/W chromosomes with few remaining genes are, however, considered poor model systems for studying the evolutionary forces and molecular mechanisms driving degeneration. This is simply because most genes vanished long ago, meaning that the relevant information has become obscured or lost over time^8,9^. Furthermore, the study-bias towards XY systems hampers our general understanding of the temporal aspect of the degenerative process, as the type of heterogamety is expected to influence the rate of degeneration^9,10^. It is predicted that Y chromosomes undergo more rapid degeneration compared to W chromosomes, due to higher mutation rates, stronger sexual selection pressures and lower effective population sizes in males than in females^10,11^. To broaden our understanding of sex chromosome evolution, we therefore need to study W chromosome degeneration in additional young sex chromosome systems.

One way to investigate the dynamics of W degeneration is to focus on “neo-sex chromosomes”^12–14^, which form through translocations of parts of autosomes to existing sex chromosomes or fusions between entire autosomes and sex chromosomes^7^. These chromosomal rearrangements are often followed by expansion of recombination suppression into the translocated region, thus initiating the degeneration process on new genetic material^7^. Neo-sex chromosomes provide an opportunity to investigate how the degeneration process proceeds through evolutionary time, since the timing of recombination suppression often varies over the chromosome and between species^7,15^. The sex chromosomes of birds, which formed > 100 million years ago (Mya), were previously thought to be extremely stable and share the same gene content across the entire clade^16,17^. Recently, however, this notion has been challenged with neo-sex chromosomes being found in several lineages^18^, including in parrots, cuckoos, crested ibis, and songbirds^19–24^.

**Figure 1:**
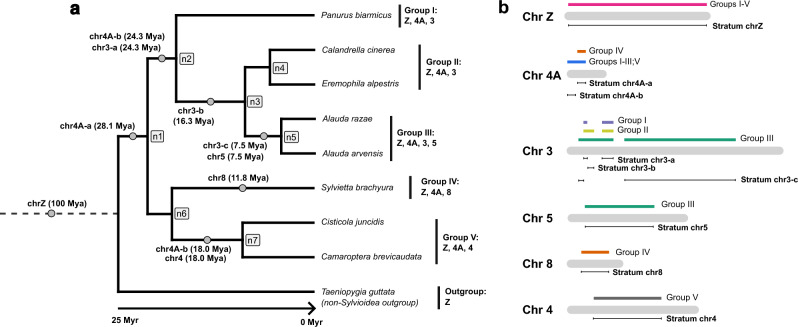
Phylogeny of the study species, and inferred formation time and genome range of all sex-linked strata. (**a**) Phylogeny of the eight studied Sylvioidea species. These are grouped (I-V) according to those sharing identical sex-linked regions, based on previous studies (see Main text). The grey dots represent the inferred time when each region became sex-linked (calculated as the branch midpoint between two dated nodes (n1-n7 in grey boxes); see Methods; Supplementary Fig. 2,5). Note that chromosome Z is the ancestral sex chromosome of birds, and is therefore also present in the outgroup species. (**b**) Differently coloured bars above each chromosome (in grey) marks the extent of sex-linkage associated with each group (I-V). We divided these sex-linked regions in strata according to shared sex-linked regions between monophyletic groups. Note that for chromosome 4A, this means that stratum chr4A-b became sex-linked twice independently (see Main text).

In Sylvioidea songbirds, there have been at least five independent translocations of parts of autosomes to the sex chromosomes, leading to the highest neo-sex chromosome diversity known in birds^23,25,26^. One of these translocations is shared between all members of the group (homologous to parts of chromosome 4A in zebra finch), while the others (homologous to parts of chromosomes 3, 4, 5 and 8, respectively) are specific to certain lineages^19,22,23,27,28^. Translocations to both Z and W are confirmed for chromosome 4A^15,26^, and highly supported for chromosomes 3 and 5^28,29^, whereas linkage to W is not yet confirmed for chromosomes 4 and 8^22,23^. However, the translocated regions are partly non-recombining^22,23,28^ and thus affected by the typical features of W chromosome degeneration, which include gene loss and repeat accumulation^15^. Here, we targeted eight Sylvioidea species from four different families which all have multiple autosome–sex chromosome translocations, i.e., at least one more than the chromosome 4A-translocation (Fig. 1), to provide the most comprehensive study of W chromosome degeneration rates to date. We also use this study system to test whether less important genes degenerate first, as has been shown for the ancient sex chromosomes of mammals and birds^8,30^ but to a lesser extent for recently evolved sex-linked regions (but see^31^). Throughout this paper, we use the term “sex-linked” to describe sex chromosome regions that are recombination suppressed and exhibit genetic differences between Z and W. Note, however, that the pseudoautosomal regions (regions with retained recombination between sex chromosome copies) are strictly speaking also sex-linked, though partially^32^.

We generated presence-absence data for W-linked gametologs (i.e., genes on the W chromosome with homologs on the Z chromosome) for all eight study species using whole-genome sequencing data of males and females. Our aims were (1) to estimate the level of W chromosome degeneration on the neo-sex chromosomes, measured by the proportion of maintained W-linked gametologs. Y/W chromosome degeneration is hypothesised to progress in a negative exponential manner, with an initial high rate of decay which levels off with fewer remaining Y/W-linked gametologs^33^. To test this prediction, we (2) inferred when each W-linked gametolog was lost using a phylogenetic approach, which allowed us to study the temporal aspect of W chromosome gene decay. Lastly, we (3) tested whether genes with a maintained W-linked gametolog differed in “importance” to those where only the Z-linked gametolog remained, using level of sequence conservation [based on non-synonymous (dN) to synonymous substitution rate (dS) ratios], and degree of haploinsufficiency, i.e., the sensitivity to the loss of one gene copy (based on haploinsufficiency scores;^34,35^), as proxies.

## Methods

We whole-genome sequenced DNA extracted from blood samples of a wild-caught male and female from eight Sylvioidea species (2 × 150 bp paired-end). Of these 16 individuals, 4 are novel for this study (Supplementary Table 1) while 12 are previously published and described^22,23,28^ (Supplementary Table 1). The experimental protocol for the non-destructive sampling was approved by the relevant authorities (Direcção Geral do Ambiente, Cape Verde, and Malmö/Lund Ethical Committee for scientific work on animals, Sweden, no. 17277-18). All methods, which were carried out according to the guidelines and regulations from these authorities, are reported in accordance with the ARRIVE guidelines for animal experiments.

To ensure that the results from the different Sylvioidea species were not affected by quality differences in genome assemblies or annotations, we used the genomic resources from the zebra finch (*Taeniopygia guttata*; taeGut.3.2.4; genome and annotation^36^) as a backbone in this study. Using the high-quality gene annotation of the zebra finch also allowed for better functional annotations of loss-of-function genetic variants (see below).

### Zebra finch gene annotation

From the zebra finch gene annotation (taeGut3.2.4.92), we extracted the longest transcript from each gene using gtf2gtf (options − method = filter − filter-method = longest-transcript) from the CGAT v0.3.3 collection of tools^37^. From this filtered GTF file, we extracted (1) the full genomic range of each gene (n = 18,618) and (2) the genomic ranges of each exon (n = 160,397). Using these two files, we also extracted the genomic ranges of each intron (n = 152,937) by extracting genomic regions occurring within the genomic range of each gene that was not covered by an exon, using the program bedtools v2.27.1 complement^38^.

### Alignment, sequencing depth, variant calling, and functional annotation of variants

Whole-genome sequence data from a female (n = 1) and a male (n = 1) sample from each of the 8 Sylvioidea species (n = 16 individuals; Supplementary Table 1) were aligned to the zebra finch genome using NextGenMap v0.5.5^39^, sorted with samtools v1.14^40^, and deduplicated with picardtools v2.18.0 (http://broadinstitute.github.io/picard). We calculated sequencing depth statistics from each exon and intron (n = 313,334) and sample (n = 16) using the program bamstat04 from the jvarkit toolbox^41^. The output from this program includes mean and median values for each specified genomic range. Using mosdepth v0.3.3^42^, we calculated the number of callable sites (defined as minimum 5× sequencing depth of uniquely mapping reads) per exon and intron. We called genetic variants within the genomic ranges of each zebra finch gene (n = 18,618; see above) using freebayes v1.3.2^43^ for each sample separately.

Each resulting VCF file was filtered using vcftools v0.1.16^44^ for minimum of one non-reference allele (-non-ref-ac 1), quality scores of 30 or higher (-minQ 30), minimum depth of 5 (-minDP 5), with quality flag PASS (-remove-filtered-all) and no missing genotypes (-max-missing 1). The genetic variants were then further filtered using the vcflib v1.0.3^45^ program vcffilter in the following way: allele balance either between 0.25 and 0.75, or lower than 0.01 (-s -f “AB > 0.25 & AB < 0.75 | AB < 0.01”), similar mapping qualities between reference and alternative alleles (-s -f “MQM/MQMR > 0.9 & MQM/MQMR < 1.05”) and between reads supporting the reference and alternative alleles (-s -f “PAIRED > 0.05 & PAIREDR > 0.05 & PAIREDR/PAIRED < 1.75 & PAIREDR/PAIRED > 0.25 | PAIRED < 0.05 & PAIREDR < 0.05”).

Using these filtered VCF files (n = 16), we annotated and predicted the effects of genetic variants on proteins using snpEff v4.3t^46^ and the already available snpEff database created from the zebra finch genome and annotation: taeGut3.2.4.86. Using snpSift v4.3t^47^, we extracted heterozygous sites (java -jar SnpSift.jar filter “(countHet() > 0)”) and loss-of-function mutations (i.e., genetic variants predicted to cause a loss of protein function) that affected more than 50% of transcripts (java -jar snpSift.jar filter “(exists LOF[*].PERC) & (LOF[*].PERC > 0.5)”) for each VCF file. The output was intersected with the genomic ranges of introns and exons (n = 313,334), and the number of heterozygous sites and loss-of-function mutations were summed for each genomic range.

### Creating a dated phylogeny

To construct a phylogenetic tree for the 8 study-species (and outgroups) we created consensus genomes from the VCF files with the male sample of each species using bcftools v1.16^48^ with option –haplotype A, incorporating only genetic variants within exon regions. We then translated the gene coordinates from the zebra finch annotation to the consensus genomes using the program liftoff v1.6.3^49^ with options -mm2_options = “-r 2k -z 5000” -polish. Using seqtk subseq (https://github.com/lh3/seqtk), we extracted the coding (CDS) regions from all genes with complete open reading frames (marked with “valid_ORF = True” in the liftoff output file) using gffread v012.7^50^.

We downloaded gene annotations, as well as protein and CDS sequences, from Ensembl Release 111^51^ from the following eight species: green anole (*Anolis carolinensis*), emu (*Dromaius novaehollandiae*), great spotted kiwi (*Apteryx haastii*), chicken (*Gallus gallus*), mallard (*Anas platyrhynchos*), budgerigar (*Melopsittacus undulatus*), blue-crowned manakin (*Lepidothrix coronata*), and collared flycatcher (*Ficedula albicollis*). The longest isoform of each gene was extracted using the script agat_sp_keep_longest_isoform.pl from the AGAT v1.3.2^52^ suite of tools, and an orthology analysis was done using OrthoFinder v2.5.2^53^. From the single-copy orthologs we selected genes from chromosomes which were autosomal across all Sylvioidea species (i.e., not Z, 3, 4, 4A, 5, or 8) and from which ORF genes had been extracted from all Sylvioidea species in this study (n = 707 genes). We aligned CDS sequences from each of these genes, containing sequences from each outgroup species (n = 9, including zebra finch) and the Sylvioidea species (n = 8), using PRANK v170427^54^ with default settings. The conserved blocks in the alignments were selected using GBLOCKS v0.91b^55^ based on codons (options: − t = c − p = n). All trimmed alignments longer than 500 bp were then concatenated and used for reconstructing the maximum likelihood (ML) species phylogeny by using RAxML v8.2.12^56^ with green anole as an outgroup (-m GTRGAMMAX -f a -p 202009 -× 202009 -N autoMRE -o anole). The topology of the ML phylogeny was extracted for later dating analysis (see below).

The 100 longest gene alignments (≥ 1923 bp) were concatenated and analysed with Baseml in PAML v4.9^57^ for calculating the gamma distribution shape priors (i.e., alpha and beta) rate for genes. Running Baseml is configured using a control file (provided in Supplementary Information). Specifically, the topology of the ML phylogeny with the following calibration times (from^58,59^): divergence of lizard–bird (255.9–299.8 Myr), Palaeognathae–Neognathae (66–99.6 Myr), Psittaciformes–Passeriformes (51.81–66.5 Myr) and suboscine–oscine passerines (27.25–56 Myr), was set to be the user tree (runmode = 0), HKY85 was selected (model = 4) and standard error (S.E.) of estimates were to be calculated (getSE = 1). In the output, we obtained the mean and S.E. for the substitution rate, based on which gamma distribution shape priors were calculated. Next, the topology of the ML phylogeny was then dated with MCMCTree in PAML v4.9^57^ with calculated gamma distribution shape priors and the same calibration times as used in the Baseml analysis. Running MCMCTree is configured with a control file (provided in Supplementary Information). The dated phylogeny was plotted using the MCMCtreeR R package (https://github.com/PuttickMacroevolution/MCMCtreeR, last accessed on June 13, 2023) and provided as Supplementary Fig. 1. Supplementary Fig. 2 show the same tree as a cladogram with all node ages as labels.

### Assessing gene loss across all species

Using R v4.2.3^60^, we populated a data frame with sex-specific values of (1) sequencing depth, (2) heterozygous sites and (3) loss-of-function sites per exon/intron and species. Extreme sequencing depth values for exons/introns (lower than 5× or higher than twice the mean sequencing depth value across all autosomes), as well as values from short exons/introns (< 50 bp callable sites), were masked. A whole-genome median sequencing depth value per species and sex was calculated from exons/introns of all chromosomes except those known to be fully or partially sex-linked in species across our dataset (chromosomes Z, Z_random, 4A, 3, 4, 5 and 8). We normalized the ratio between the female and male value for each unmasked exon/intron by dividing it with the whole-genome female-to-male ratio. Normalized sequencing depth ratios above 2 or below 0.1 were masked (depth distributions before and after normalization are given in Supplementary Figs. 3,4). We divided the number of heterozygous sites per exon/intron with the number of callable sites, for each species and sex, to get a proportion of heterozygous sites and calculated the difference between the female and male value per species.

We calculated the mean and standard deviation of (1) female-to-male sequencing depth ratio and (2) female-to-male difference in heterozygosity proportion for each gene from the exon and intron values. We also counted the number of (1) female-specific and (2) male-specific loss-of-function mutations occurring within exon regions of each gene. We removed genes from chromosomes labelled as “random” (concatenated scaffolds known to belong to a certain chromosome which could not be confidently placed during the genome assembly process) or “Un” (concatenated scaffolds of unknown chromosomal origin). Lastly, we removed genes with extreme sequencing depth values (see above), genes where both sexes had loss-of-function mutations in exons, and filtered the dataset for genes where the cumulative length of exons and introns were over 700 bp (leaving n = 10,628 genes).

The genomic ranges of sex-linked (i.e., non-recombining) regions for the studied species are known from previous studies (^22,23,28^ Supplementary Table 2). For each gene and species, we added information on whether each gene is positioned within a sex-linked genomic region. Next, we divided genes into categories of being either (1) diploid in the female (i.e., gene is present on both the Z and W chromosome; referred to in this paper as “W-maintained”) or (2) haploid in the female (i.e., gene is missing from the W chromosome; referred to as “W-lost”), based on their combined sequencing depth and heterozygosity characteristics. Genes with female-to-male sequencing depth ratios > 0.75 were labelled as “W-maintained”. Genes where the female-to-male sequencing depth ratio was < 0.70 and the heterozygosity value was < 0.001, or where the female had a loss-of-function mutation, were classified as “W-lost” in females. The remaining genes (with female-to-male sequencing depth between 0.7 and 0.75, or with female-to-male sequencing depth < 0.75 and heterozygosity > 0.001, without female loss-of-function mutations) were labelled as “unsure”.

After filtering, between 8920 and 9802 genes remained across the species, of which 755–1642 were positioned on a sex-linked region in each species. Across the species, between 0.2 and 0.9% genes were labelled as “W-lost” despite not being on a sex-linked genomic region in these species. In contrast, between 36.0 and 72.8% of genes within sex-linked regions were labelled as “W-lost”. Finally, we excluded all genes that were missing in one or several species, and those occurring in genome regions which are autosomal in all species (leaving n = 1931 genes).

### Inferring the timing of gene loss across the phylogeny

To determine the timing of gene loss for all sex-linked genes, we used the dated phylogenetic tree of all the included species (see above) and inferred, starting from the tips of the tree, whether W genes were present or absent at each node (n1–n7; Fig. 1; Supplementary Fig. 5). Specifically, if a gene was scored as “W-maintained” in either, or both, of two sister-species it was categorized as present on the W chromosome at the node connecting these two species. If a gene was scored as “W-lost” in both sister-species, it was categorized as lost at the node connecting these species. Following the same logic, we then scored genes as present (or lost) at internal nodes if it was present (or lost) in either, or both, descendent nodes (see Supplementary Fig. 6). Note that these estimates capture the minimum age of gene loss, and that the actual gene loss would have been somewhere along the branch leading up to the node in question.

### Rate of gene loss

We subset the data further to estimate the rate of gene loss. Firstly, we filtered the data to only include genes from sex-linked regions in each species. We then calculated the total number of sex-linked genes (from all strata) estimated to be present at each node. To determine the rate of gene loss independently of subsequent fusions, which may be expected to change the dynamics of gene loss, we focused only on nodes where all strata had been added for each species (Supplementary Fig. 7). In cases where sister-species shared the same strata, we selected the first one in the phylogeny to avoid pseudo-replication in our statistical analysis. Note that this does not bias our results since the sister species in our sample have almost identical gene content. Specifically, we extracted gene loss values from four different lineages: (1) node2 to *Panurus biarmicus* (n2-Pbia), (2) node3 to *Calandrella cinerea* (n3-Ccin), (3) node5 to *Alauda arvensis* (n5-Aarv), and (4) node6 to *Cisticola juncidis* (n6-Cjun; Fig. 1; Supplementary Fig. 7).

To estimate the absolute rate of gene loss, we divided the number of genes lost in each lineage by the age of the node (extracted from the dated phylogeny; Supplementary Figs. 2,5). We did this separately for each of the four lineages, and we also calculated the average of these values. We then calculated the proportional rate of gene loss, by dividing the number of genes lost by the total number of genes present at the starting node multiplied by 100, divided by node age. Again, we did this for each lineage separately and calculated the average of these values.

### Identification of dosage sensitive genes and outgroup dN/dS values

We downloaded one2one orthologs between zebra finch and chicken from Ensembl BioMart^51^, including information about gene-specific substitution rates (dN and dS). From Ensembl, we also downloaded one2one orthologs between zebra finch and human (including gene symbols). We cross-referenced the human-zebra finch orthologs with downloaded human haploinsufficiency (HI) scores from the DECIPHER database^34,35^. HI scores describe the likelihood of genes being found in a haploid state based on results from medical studies and is commonly used as a proxy for dosage sensitivity. Genes with high HI scores are more likely to be sensitive to dosage changes than those with low HI scores. The dN/dS and HI scores were then cross-referenced on the presence-absence data for the Sylvioidea W chromosomes (n = 1931 genes), using the zebra finch transcript ID’s.

To avoid pseudo-replication and the confounding effects of subsequent fusions on gene loss dynamics, we only examined the genes used to quantify the rate of gene loss in the four lineages described above (n2-Pbia, n3-Ccin, n5-Aarv and n6-Cjun). Genes present at the starting node which are still present in each species were scored as present; those lost within each species were scored as absent. For each lineage we then tested if the probability of W gene loss (binary response variable) depended on the (1) HI scores and (2) dN/dS values between outgroup bird species by fitting logistic regression models (glm) in R v4.2.3^60^. Since we modelled HI scores and dN/dS separately, we constructed eight models in total (i.e., two for each of the four lineages). We also constructed two random regression models (glmer) based on the pooled data from the four lineages. In each model the presence/absence of each gene was the response variable and either HI score or dN/dS was the fixed effect. We specified a random effect structure to allow each lineage to have its own slope and intercept. This allowed us to estimate the overall slope and intercept while accounting for the variance between lineages in these parameters.

## Results

### Defining strata on the Sylvioidea neo-sex chromosomes

The precise genomic regions that are sex-linked in each studied species are summarized in Fig. 1 (based on previous studies). We grouped the studied species according to those which share identical sex-linked genomic regions (Group I-V; Fig. 1a,b; Supplementary Table 2). We then divided these sex-linked regions into different “strata”, by identifying the genome regions shared by monophyletic groups which ceased to recombine in a common ancestor (Fig. 1a,b; Supplementary Table 2). This division is partially supported by results from a previous study, which showed that the rate of synonymous substitutions (dS) between gametologous gene pairs positioned on what we define here as strata chr3-a, chr3-b, and chr3-c correlated significantly with this relative order of recombination suppression^28^. Note that by this definition, stratum chr4A-b ceased to recombine twice independently, as it is still recombining in *Sylvietta brachyura* (Fig. 1a).

This division into strata allowed us to compare W degeneration rates between regions, although it is most likely an oversimplification as recombination suppression may have formed by more gradual processes (and also independently; see^15,61^). The estimated ages of the seven phylogenetic nodes within Sylvioidea (n1-n7; Fig. 1) ranged between 26.4 Mya (n1; Supplementary Figs. 2,5) to 4.5 Mya (n5; Supplementary Figs. 2,5). As we do not know the exact time of the autosome–sex chromosome translocations, we infer that each translocation occurred at the midpoint of the branch leading up to the first node (internal or terminal) where it is present in all descendent species (Fig. 1a; Supplementary Fig. 5). For the ancestral sex chromosome (chrZ), we used the estimated origin age from Zhou et al.^62^ of 100 Myr (Fig. 1a). Note, however, that the evolutionary history of the ancestral sex chromosome is more complex than this, with independent formation of at least four strata, possibly formed through inversion events (see e.g.,^63–65^).

### Proportion of lost vs present genes

**Figure 2:**
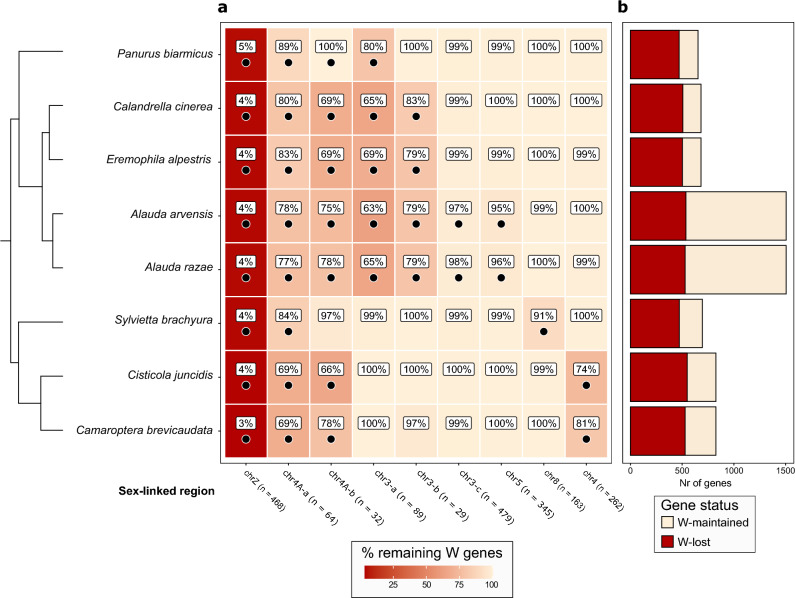
Lost versus maintained W gene content in all study species and strata, based on presence-absence of genes in the eight species. (**a**) Proportion of maintained W-linked gametologs for each stratum. Species where each stratum is sex-linked are indicated with a black dot inside the tile, while strata that are not sex-linked lack dots. The colour shade corresponds to the values inside the tiles. The numbers next to chromosome names (e.g. “chrZ (n = 468)”) indicate the original number of genes in each strata. (**b**) Number of maintained versus lost W genes in each species.

After filtering, the number of genes in the different strata ranged between 29 and 479 (Fig. 2). Only 3.4–4.7% of the W-linked gametologs on the ancestral sex chromosome were maintained across the studied species (chrZ; Fig. 2a; mean across species ($\overline{x }$): 4.2%). On the more recently added strata, only considering species where each stratum is sex-linked, these values ranged between 62.9% and 100.0% ($\overline{x }$: 83.6%; Fig. 2a). The region on chromosome 4A that is sex-linked across all Sylvioidea species, and inferred to be the second oldest stratum (chr4A-a), had 68.8–89.1% maintained W-linked gametologs (Fig. 2a; chr4A-a; $\overline{x }$: 78.5%). The second stratum on chromosome 4A (chr4A-b), which is shared by all studied species except for *Sylvietta brachyura*, had more variation between species with values ranging between 65.6 and 100.0% (Fig. 2a; chr4A-b; $\overline{x }$: 76.3%). The three strata on chromosome 3 (chr3-a,b,c) had values ranging between 62.9 and 97.9%, with less maintained W-linked gametologs on the stratum inferred to be oldest (chr3-a: 62.9–79.8%; $\overline{x }$: 68.3%), intermediate numbers for the stratum of intermediate age (chr3-b: 79.3–82.8%; $\overline{x }$: 80.2%), and many maintained gametologs on the youngest stratum (chr3-c: 97.5–97.9%; $\overline{x }$: 97.7%; Fig. 2a). The stratum on chromosome 5 (chr5; 95.4–96.2, $\overline{x }$: 95.8%) had similar values as those of stratum chr3-c, in line with previous results showing a similar level of Z-to-W divergence for these regions^28^. The proportion of maintained W-linked gametologs in strata chr3-c and chr5 overlapped with the distribution from autosomal values, suggesting extremely little W chromosome degeneration (Fig. 2a). The stratum on chromosome 8 appeared less degenerated (chr8; 90.8% maintained W-linked gametologs) than the stratum on chromosome 4 (chr4; $\overline{x }$: 74.4–80.9, 77.7%; Fig. 2a).

We found pronounced between-species variation in the proportion of maintained W-linked gametologs within some strata. This variation was most notable at strata chr4A-a and chr4A-b (Fig. 2a), where *Panurus biarmicus* showed a higher proportion of maintained W-linked gametologs (89.1 and 100.0%, respectively) compared to other species (68.8–84.4 and 65.6–78.1%, respectively). The total number of sex-linked genes (with and without W-linked gametologs) ranged between 653 and 1,506 across the species, with the two *Alauda* species (both 1,506) having ~ twice the number of sex-linked genes than the rest as a consequence of their multiple translocations of large chromosome regions to the sex chromosomes ($\overline{x }$: 727; Fig. 2b). However, the number of W genes lost was similar between species ($\overline{x }$: 512; Fig. 2b).

### Inferring the timing of W gene loss

**Figure 3:**
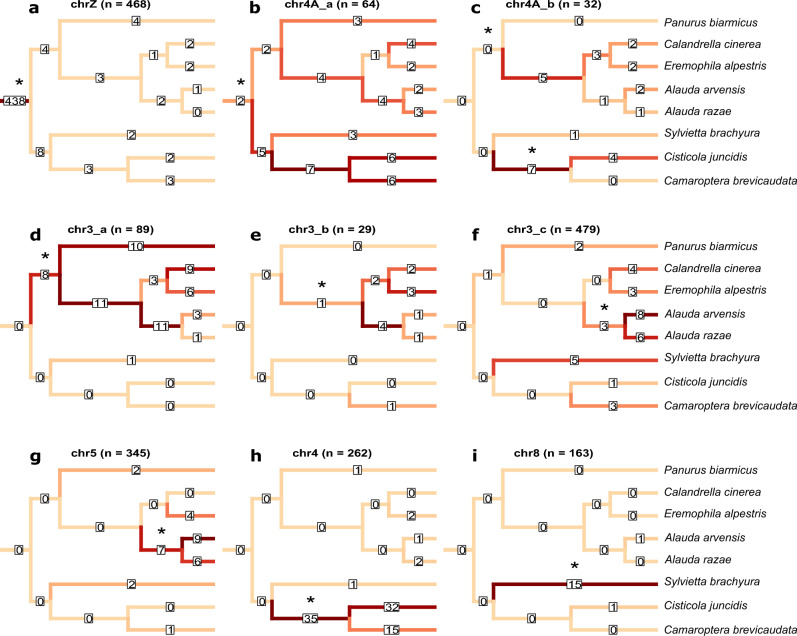
Estimated W gene loss on strata plotted on phylogenetic trees, for each stratum (**a**–**i**). The branches are coloured (pale yellow to dark red indicating few to many lost genes) and labelled (numbers in white boxes) by the number of W genes lost at their adjacent node (towards the leaves of the tree). The asterisks mark the position in the tree where each region became sex-linked. Corresponding numbers for all strata (**a**–**i**) are provided in Supplementary Table 3.

Based on the W status of individual genes across species (“W-maintained” and “W-lost”), we reconstructed the likely timing of W gene loss across the phylogeny. The same inferences were made for all species across all strata, regardless of which regions were sex-linked in each species. This allows us to evaluate the error rate of this method, as we are not expecting genes on autosomal regions to be classified as “W-lost”. These analyses show that most W-linked gametologs on the ancestral sex chromosome (Fig. 3a; chrZ; 93.6%; 438/468) were lost prior to the formation of the Sylvioidea clade (26.4 Mya; Supplementary Fig. 2). However, all nodes except one (the *A. razae* terminal node) were associated with at least one (1–4) additional loss of W-linked gametologs, meaning that W chromosome degeneration on this old stratum is ongoing (Fig. 3a).

For the sex-linked region on chromosome 4A that is shared by all studied species (chr4A-a), only 3.1% (2/64) of the original genes were lost ancestrally in the Sylvioidea clade (Fig. 3b). This is expected as the Sylvioidea families we are studying are basal to the group and radiated early after its formation^59^. Most W-linked gametologs from this stratum have therefore been lost at internal nodes within Sylvioidea. All other sex-linked regions, which were formed after speciation events between the studied species, showed 0.0% W gene loss at the branch leading up to all Sylvioidea species (Fig. 3c-i; Supplementary Table 3; indicating low error rates of the method). On those regions, most of the estimated W gene loss was traced to the individual species where these strata are sex-linked, and to nodes connecting only these species (Fig. 3c-i; Supplementary Table 3).

### Characteristics of lost W genes, and the rate of W chromosome degeneration

As species share branches in the phylogeny, which violates statistical independence, and as subsequent fusions may change the dynamics of gene loss, we used data from four independent nodes not affected by additional translocations (see Methods; Supplementary Fig. 7) to estimate the rate of W gene loss (Table 1), and to test whether the probability of gene loss depended on being evolutionary constrained (measured using dN/dS values; Table 2) and/or dosage sensitive (measured using HI scores; Table 3). The absolute rate of W gene loss varied between 0.8 and 6.0 genes per million years (Myr) across these four Sylvioidea lineages ($\overline{x }$: 3.4 genes/Myr; Table 1). The proportional rate of W gene loss ranged between 0.4 and 1.6% lost genes per million years ($\overline{x }$: 1.0% gene loss/Myr; Table 1). Neither the absolute rate nor the proportional rate of gene loss showed any consistent association with the age of the strata or with the number of W genes at the formation of the strata (Table 1).

**Table 1:** The rate of W gene loss across four independent lineages.

Lineage	Node age (Myr)	No. of W genes at node	No. of W genes lost	W gene loss/Myr	% W gene loss	% W gene loss/Myr
n2-Pbia	22.2	190	17	0.8	9.0	0.4
n3-Ccin	10.4	177	30	2.9	1.7	1.6
n5-Aarv	4.5	1013	27	6.0	2.7	0.6
n7-Cjun	12.3	286	46	3.7	16.1	1.3
Mean across lineages				3.4	7.4	1.0

(n2-Pbia, n3-Ccin, 5-Aarv, and 7-Cjun; see Main text and Fig. 2).

For each lineage, the table shows the (1) age of the starting node in Myr, (2) the number of W genes remaining at this node, (3) the number of lost W genes, (4) the number of W genes lost per Myr, (5) the percentage of W genes remaining compared to the starting node and (6) the percentual loss of W genes per Myr. The last line shows mean values of the lineage-specific values.

**Table 2:** Results from general linear models for each of the four lineages (glm) and a random regression model for pooled data of all four lineages (glmer) to test if the dN/dS values (proxy for evolutionary constraint) predict the probability that a W gene present at the starting node in each lineage is lost.

Lineage	Node age (Myr)	No. genes at node	No. genes lost	Test	Estimate [log odds]	Std. Err	z value	*p* value	Sig
n2-Pbia	22.2	143	14	glm	− 5.8	3.0	− 1.9	0.06	–
n3-Ccin	10.4	137	22	glm	− 5.1	2.3	− 2.2	< 0.05	*
n5-Aarv	4.5	820	20	glm	− 4.1	1.6	− 2.6	< 0.05	*
n7-Cjun	12.3	205	34	glm	− 3.4	1.2	− 2.9	< 0.01	**
Lineages pooled				glmer	− 3.9	0.8	− 4.7	< 0.001	***

See Methods for details.

**Table 3:** Results from general linear models for each of the four lineages (glm) and a random regression model for pooled data of all four lineages (glmer) to test if the HI scores (proxy for dosage sensitivity) predict the probability that a W gene present at the start node in each lineage is lost.

Lineage	Node age (Myr)	No. genes at node	No. genes lost	Test	Estimate [log odds]	Std. Err	z value	*p* value	Sig
n2-Pbia	22.2	133	11	glm	− 0.03	0.01	− 2.3	< 0.05	*
n3-Ccin	10.4	129	20	glm	− 0.04	0.01	− 3.6	< 0.001	***
n5-Aarv	4.5	778	19	glm	− 0.02	0.01	− 2.7	< 0.01	**
n7-Cjun	12.3	204	31	glm	− 0.03	0.01	− 3.3	< 0.001	***
Lineages pooled				glmer	− 0.03	0.01	− 5.5	< 0.001	***

See Methods for details.

The probability of W gene loss was significantly negatively correlated with dN/dS values in three of the four lineages (*p* < 0.06–0.01) and when these lineages were pooled (*p* < 0.001), suggesting that genes under purifying selection are disproportionally maintained on the W chromosomes (Table 2). The HI scores also significantly affected the probability of gene loss in each of the four lineages (*p* < 0.05–0.001) and when these lineages were pooled (*p* < 0.001), with haploinsufficient genes (i.e., genes sensitive to the loss of one gene copy) being maintained to a higher degree than others (Table 3). These results are in line with previous studies^8,30,31^.

## Discussion

We show that the different sex-linked strata identified in eight Sylvioidea species with partly different sex-linked chromosome regions exhibit the full range of W chromosome degeneration stages. The ancestral sex chromosome (which we here treat a single stratum, chrZ) had an overall low proportion of maintained W-linked gametologs (4.2%), while the genomic regions that have become sex-linked through translocations to the ancestral sex chromosome within Sylvioidea (all except chrZ) exhibited a wide range of W degeneration stages, from intermediate levels (68.3–90.8% maintained genes on chr4A-a, chr4A-b, chr3-a, chr3-b, chr4 and chr8) to almost no degeneration (95.8–97.7%) on chr3-c and chr5. As expected, older strata had overall lost a higher proportion of genes. The variance in degeneration between strata was largest in the two *Alauda* species (*A. arvensis* and *A. razae*), which both display the full spectrum of W chromosome degeneration stages on their many strata (chrZ, chr4A-a,b, chr3-a,b,c, and chr5; Fig. 2). The proportion of maintained genes on the ancestral W chromosome are concordant with studies from other birds, partly because many of these genes were lost deep in the avian phylogeny. For example, the W chromosome in chicken (*Gallus gallus*) was estimated to have 4.0% maintained W-linked gametologs (28 genes out of 685 original genes;^30^). Another study found 46 W-linked gametologs maintained in the collared flycatcher (*Ficedula albicollis*;^66^), which would correspond to 6.7% based on the same number of original genes as estimated in chicken (n = 685;^30^). We found that most of the W genes on the ancestral sex chromosome (Fig. 3) became lost prior to the formation of Sylvioidea (438 of the original 468 genes; 93.6%), and that 17 genes (3.6% of the original gene content) were lost at least once within Sylvioidea (i.e., during the last 26.4 Myr). These results show that the ancestral W chromosome is still experiencing significant gene loss in birds, in line with recent results across other passerines^67^. In contrast, empirical studies have shown that there has been almost no gene loss on the already heavily degenerated mammalian Y chromosome during the last 40 million years^8,68^.

To avoid pseudo-replication, we evaluated the rate of gene decay at strata in four independent lineages where no subsequent fusions occurred (as additional fusions may change the dynamics of gene loss). In those lineages, we estimated that on average 3.4 genes were lost per million years across lineages, corresponding to 1.0% gene loss per million years when accounting for the variation in the number of genes at the time of strata formation of each lineage (Table 1). This rate of gene loss is lower than on the neo-sex chromosome found among parrots, where only 16.9% of the genes remain on the added part after 31.8 Myr of evolution (corresponding to a 2.6% gene loss/Myr;^20^), but higher than that of the neo-sex chromosome in the crested ibis, where only 20% were lost^24^ after ~ 53 Myr (^62^; age according to strata S3), corresponding to a gene loss rate of 0.4 genes/Myr. Our study is based on short-read data whereas the parrot and ibis studies utilize data from assembled genomes^20,24^. A potential risk of using short-read data is incorrect assignment of SNPs as W-linked when they actually represent Z polymorphisms, thereby overestimating the rate of W degeneration. However, the degree of Z polymorphisms will be much lower than the Z–W divergence except for the very early stages of W degeneration. As our youngest stratum is > 4.5 Myr it is unlikely that misinterpreted Z polymorphisms would lead to misclassification of W genes as present or lost, which is our focus here. On the other hand, assembling the W chromosome in the heterogametic sex (ZW) carries the risk of collapsing gametologous genes that are highly similar, such as those found on recently evolved neo-sex chromosomes, which can result in varying assembly qualities and completeness across species; thus, inducing unforeseen variation in the rate of gene loss between species. Furthermore, a benefit of using short-read data is that high molecular weight DNA is not a necessary requirement, allowing the inclusion of many species in comparative genome studies. The method we have used therefore enables comparative studies and quantifying gene loss across the tree of life.

Theory predicts that genes on Y/W chromosomes will experience exponential decay over time, so that young Y/W chromosomes with many genes will experience high decay rates^7^. We found that the rates of W gene decay ranged between 0.8 and 6.0 (0.4–1.6%) genes per million years across four independent Sylvioidea lineages. In contrast to expectation^7^, the rate of gene loss was associated neither with age of the strata nor with the number of sex-linked genes at the time of strata formation in this data (Table 1). However, as we only evaluated the number of genes at two time points for each stratum in this analysis, we cannot evaluate whether the gene decay follows an exponential pattern within strata. An aim for future studies is therefore to increase the density of taxonomic sampling in the Sylvioidea tree, through which we could obtain more precise estimates of the timing of gene loss within and between strata.

At least three non-mutually exclusive evolutionary processes are hypothesized to cause Y/W gene decay, and their dynamics are expected to vary over time. These dynamics are influenced by the number and proportion of remaining Y/W-linked gametologs, as well as the effective population size of Y/W chromosomes^33^. According to Muller’s ratchet, degeneration is driven by the stochastic loss of chromosomes, including those carrying the smallest number of deleterious mutations, in a finite population^69^. According to background selection, degeneration happens as the effective population size of Y/W chromosomes decreases due to selection against strongly deleterious mutations^5^, which in turn lowers the efficiency of selection at other loci and increases drift (and Muller’s ratchet). These two processes are predicted to be particularly important for Y/W degeneration at small population sizes and on recently sex-linked, gene-rich chromosomes, with decreasing importance over time as the remaining Y/W-linked gametologs become fewer. According to genetic hitchhiking, degeneration is driven by selection for beneficial mutations which in turn increases the frequency of deleterious mutations at other linked loci^70^. Genetic hitchhiking is expected to be most influential on chromosome regions of intermediate age (but is less affected by the number of genes) and in large populations (with little drift)^33^.

Our results clearly show that purifying selection has been acting on W-linked genes in a non-random manner, and that evolutionarily constrained (i.e., genes with low dN/dS ratio) and haploinsufficient genes (i.e., genes that are sensitive to the loss of one of copy) are maintained to a higher degree than others (Tables 2,3). Selective maintenance of Y/W-linked gametologs of genes with these properties has been shown before in mammals^8^, birds^15,30,67^, and fish^31^. This suggests that selection against deleterious mutations is an ongoing component of W chromosome evolution in Sylvioidea and other taxa, which is in line with background selection as a process for W degeneration. This does not however rule out the possible importance of the other processes for W gene decay. For example, Muller’s ratchet may have been more prominent during periods of population contractions, and genetic hitchhiking more influential at strata adding relatively few genes to the non-recombining region and as the degeneration process progresses (cf.^33^).

In conclusion, our study of W degeneration in Sylvioidea songbirds, characterised by multiple autosomal translocations and numerous recombination suppression events over the past ~ 28.1 to 4.5 million years, shows that the translocated regions have maintained 68.3–97.7% of their original gene content, in stark contrast to the mere 4.2% maintained on the significantly older ancestral W chromosome. By superimposing the losses of W-linked genes onto a dated phylogeny, we estimated that the average rate of W gene loss stands at ~ 1.0% per million years. Consistent with theoretical expectations, we observed a preferential maintenance of evolutionarily constrained and haploinsufficient genes. These findings strongly indicate an ongoing role for purifying selection in countering the accumulation of deleterious mutations during W chromosome evolution in Sylvioidea. Our study provides a rare account on the dynamics of gene loss on non-recombining sex chromosomes.

### Supplementary Information

Supplementary Figures.

Supplementary Tables.

## Data Availability

All sequence data used for this study is available on NCBI (see accession ID’s in Supplementary Table 1). Code for running all analyses are available on GitHub (https://github.com/hsigeman/w-degeneration-scirep). Supplementary Figs. 1–7 are in the Supplementary Information.
