# Supplementary material for: The rate of W chromosome degeneration across multiple avian neo-sex chromosomes
Source: Sci Rep. 2024 Jul 17;14:16548. doi: 10.1038/s41598-024-66470-7 (PMC11255319; doi:10.1038/s41598-024-66470-7)
Supplement: Supplementary file 1 — Supplementary Figures. [file 41598_2024_66470_MOESM1_ESM.pdf]

SUPPLEMENTARY INFORMATION

The rate of W chromosome degeneration across multiple avian neo-sex chromosomes

BASEML AND MCMCTREE CONTROL FILES
SUPPLEMENTARY FIGURES 1-7

Baseml control file

```
seqfile = concatenation_mod.phy
treefile = RAxML_bestTree.concatenation_raxml_topology.tre

outfile = mlb       * main result file
noisy = 3   * 0,1,2,3: how much rubbish on the screen
verbose = 1   * 1: detailed output, 0: concise output
runmode = 0   * 0: user tree;  1: semi-automatic;  2: automatic
          * 3: StepwiseAddition; (4,5):PerturbationNNI

model = 4   * 0:JC69, 1:K80, 2:F81, 3:F84, 4:HKY85
        * 5:T92, 6:TN93, 7:REV, 8:UNREST, 9:REVu; 10:UNRESTu

Mgene = 0   * 0:rates, 1:separate; 2:diff pi, 3:diff kapa, 4:all diff

clock = 1   * 0:no clock, 1:clock; 2:local clock; 3:CombinedAnalysis
fix_kappa = 0   * 0: estimate kappa; 1: fix kappa at value below
kappa = 5  * initial or fixed kappa

fix_alpha = 0   * 0: estimate alpha; 1: fix alpha at value below
alpha = 0.5   * initial or fixed alpha, 0:infinity (constant rate)
Malpha = 0   * 1: different alpha's for genes, 0: one alpha
ncatG = 5   * # of categories in the dG, AdG, or nparK models of rates
nparK = 0   * rate-class models. 1:rK, 2:rK&fK, 3:rK&MK(1/K), 4:rK&MK

nhomo = 0   * 0 & 1: homogeneous, 2: kappa for branches, 3: N1, 4: N2
getSE = 1   * 0: don't want them, 1: want S.E.s of estimates
RateAncestor = 0   * (0,1,2): rates (alpha>0) or ancestral states

Small_Diff = 7e-6
cleandata = 1  * remove sites with ambiguity data (1:yes, 0:no)?
method = 0  * Optimization method 0: simultaneous; 1: one branch a time
```

MCMCTree control file

```
seed = -1
seqfile = concatenation_mod.phy
treefile = RAxML_bestTree.concatenation_raxml_topology_mod.tre
mcmcfile = mcmc.txt
outfile = out.txt

ndata = 1
seqtype = 0    * 0: nucleotides; 1:codons; 2:AAs
usedata = 1    * 0: no data; 1:seq like; 2:normal approximation; 3:out.BV (in.BV)
clock = 2    * 1: global clock; 2: independent rates; 3: correlated rates

model = 4    * 0:JC69, 1:K80, 2:F81, 3:F84, 4:HKY85
alpha = 0    * alpha for gamma rates at sites
ncatG = 5    * No. categories in discrete gamma

cleandata = 1    * remove sites with ambiguity data (1:yes, 0:no)?

BDparas = 1 1 0.1   * birth, death, sampling
kappa_gamma = 6 2      * gamma prior for kappa
alpha_gamma = 1 1      * gamma prior for alpha

rgene_gamma = 201 2309 1 0  * gammaDir prior for rate for genes
sigma2_gamma = 1 100 1   * gammaDir prior for sigma^2     (for clock=2 or 3)

finetune = 1: .05 .05 .05 .05 .05 .05 * auto (0 or 1): times, musigma2, rates, mixing, paras, FossilErr

print = 2   * 0: no mcmc sample; 1: everything except branch rates 2: everything
burnin = 100000
sampfreq = 50
nsample = 20000
```

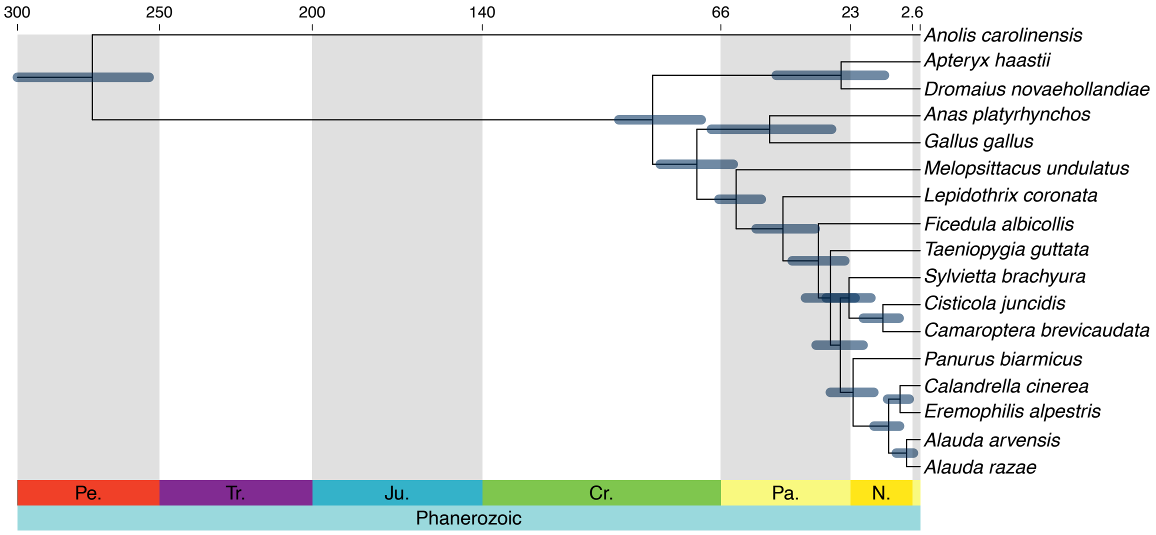

Supplementary Figure 1. Dated phylogenetic tree.

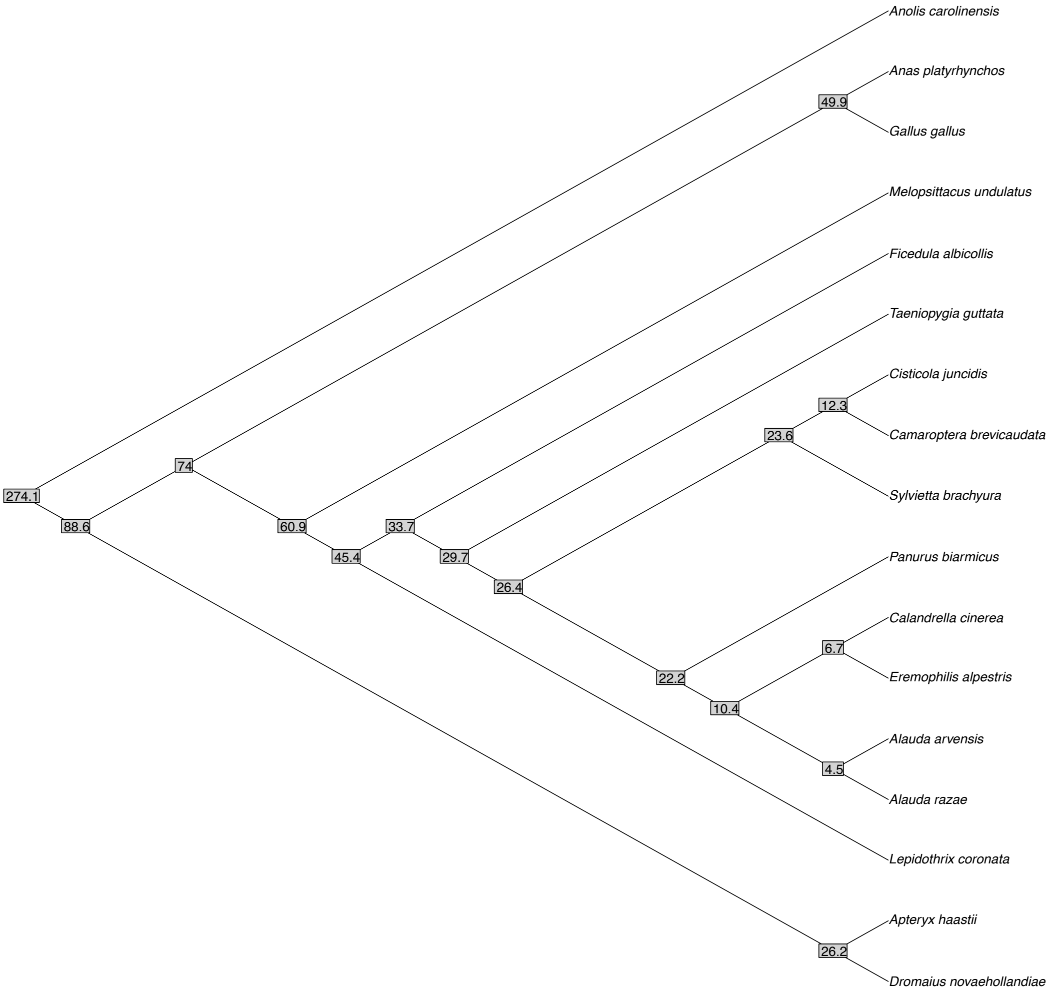

Supplementary Figure 2. Dated phylogenetic tree, shown as a cladogram with node ages (Mya) in grey boxes.

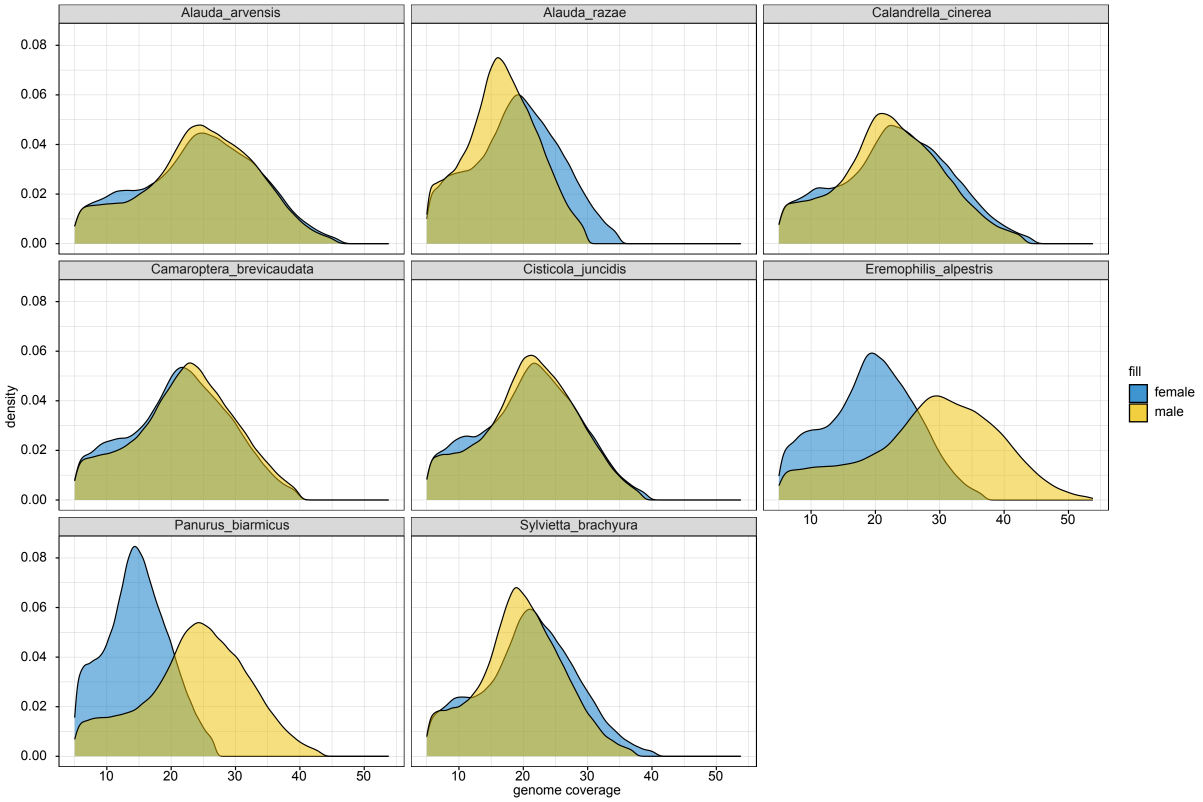

Supplementary Figure 3. Non-normalized genome coverage values for the male and female sample of each study species.

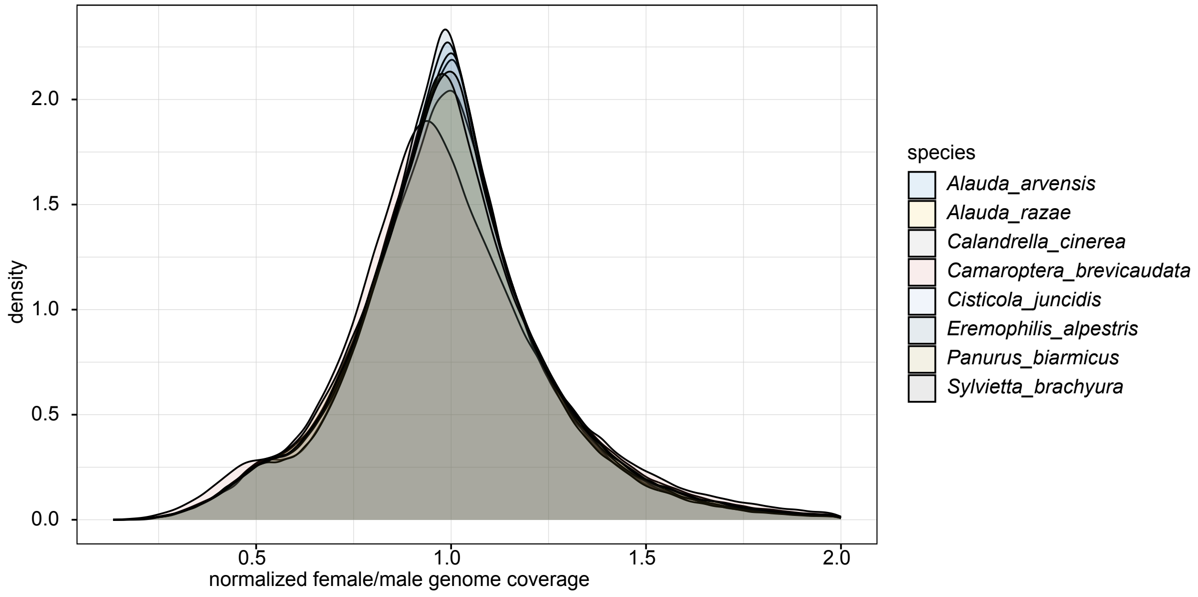

Supplementary Figure 4. Normalized female/male genome coverage ratios

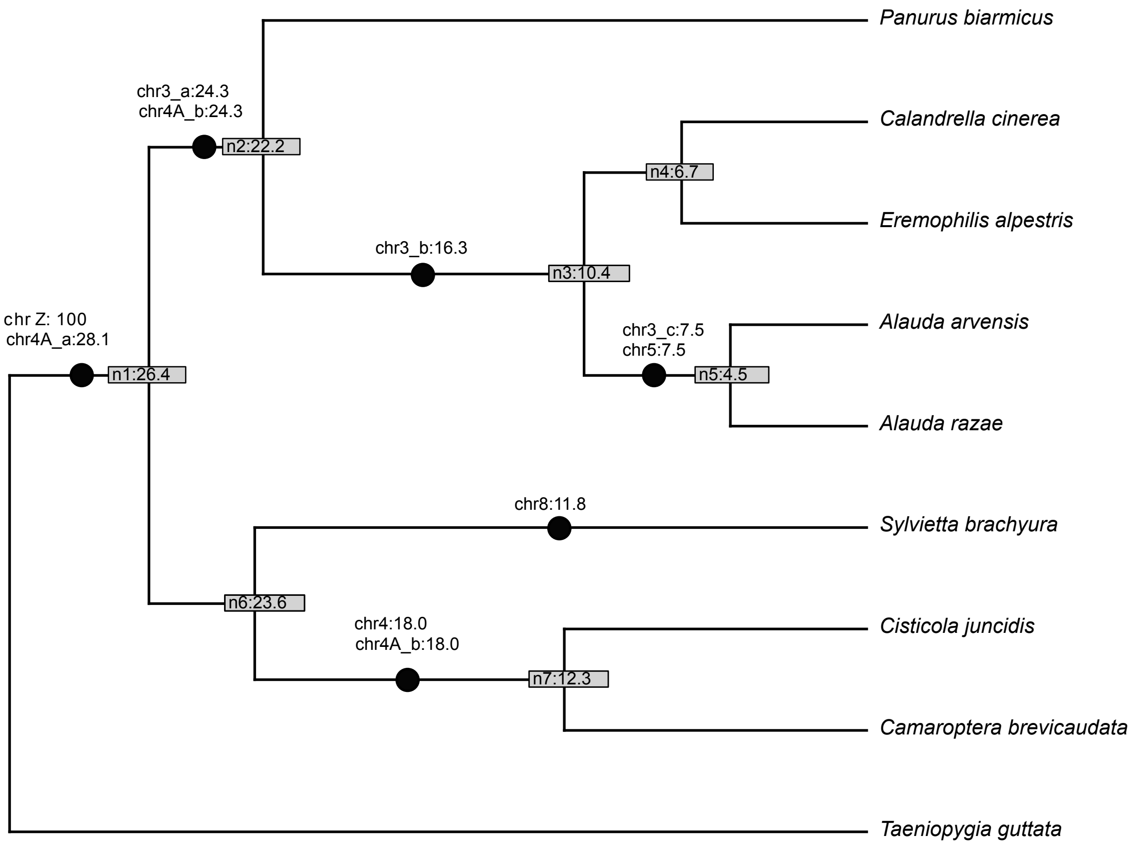

Supplementary Figure 5. Phylogenetic tree of the eight study species, and the outgroup (*T. guttata*). Internal nodes are labelled as n1-n7 (in grey boxes), with node ages (in Myr) identical to those in Supplementary Figure 2. The midpoint of branches where each stratum became sex-linked (Figure 1) is marked with a black circle. The age of each translocation event is estimated to be the midpoint of each of these branches.

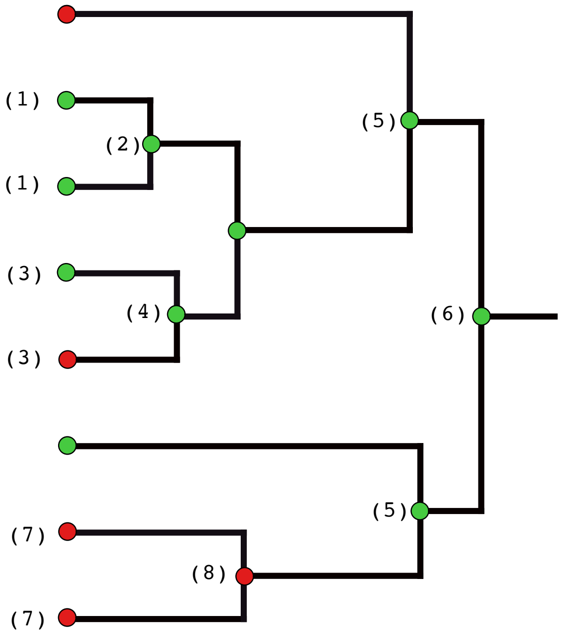

(1) W gene "present" in two sister-species = (2) gene "present" at connecting node
(3) W gene "present" in one of two sister-species = (4) gene "present" at connecting node
(5) W gene "present" at external node = (6) gene "present" at internal node
(7) W gene "missing" from two sister-species = (8) gene "missing" at connecting node

Supplementary Figure 6. Method of scoring W genes as "present" (1) or "missing" (0) at nodes based on presence-absence data at phylogenetic tips.

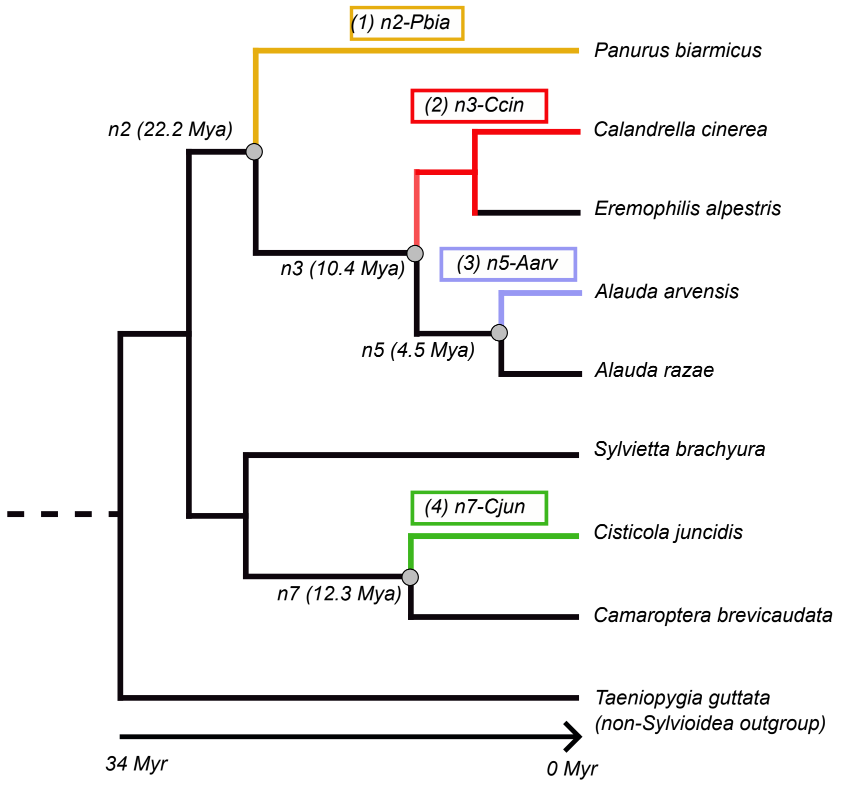

Supplementary Figure 7. Phylogenetic tree of study species showing the four independent lineages (1) "n2-Pbia", (2) "n3-Ccin", (3) "n5-Aarv" and (4) "n7-Cjun".
